# A Self-Propagating Matrix Metalloprotease-9 (MMP-9) Dependent Cycle of Chronic Neutrophilic Inflammation

**DOI:** 10.1371/journal.pone.0015781

**Published:** 2011-01-13

**Authors:** Xin Xu, Patricia L. Jackson, Scott Tanner, Matthew T. Hardison, Mojtaba Abdul Roda, James Edwin Blalock, Amit Gaggar

**Affiliations:** 1 Division of Pulmonary, Allergy and Critical Care Medicine, Department of Medicine, University of Alabama at Birmingham, Birmingham, Alabama, United States of America; 2 Gregory Fleming James CF Center, University of Alabama at Birmingham, Birmingham, Alabama, United States of America; 3 Department of Pathology, University of Alabama at Birmingham, Birmingham, Alabama, United States of America; 4 Division of Pharmacology and Pathophysiology, Utrecht Institute for Pharmaceutical Sciences, Utrecht University, Utrecht, The Netherlands; 5 Pulmonary Service, Birmingham VA Medical Center, Birmingham, Alabama, United States of America; Ludwig-Maximilians-Universität München, Germany

## Abstract

**Background:**

Chronic neutrophilic inflammation is a poorly understood feature in a variety of diseases with notable worldwide morbidity and mortality. We have recently characterized N-acetyl Pro-Gly-Pro (Ac-PGP) as an important neutrophil (PMN) chemoattractant in chronic inflammation generated from the breakdown of collagen by the actions of MMP-9. MMP-9 is present in the granules of PMNs and is differentially released during inflammation but whether Ac-PGP contributes to this ongoing proteolytic activity in chronic neutrophilic inflammation is currently unknown.

**Methodology/Principal Findings:**

Utilizing isolated primary blood PMNs from human donors, we found that Ac-PGP induces significant release of MMP-9 and concurrently activates the ERK1/2 MAPK pathway. This MMP-9 release is attenuated by an inhibitor of ERK1/2 MAPK and upstream blockade of CXCR1 and CXCR2 receptors with repertaxin leads to decreased MMP-9 release and ERK 1/2 MAPK activation. Supernatants obtained from PMNs stimulated by Ac-PGP generate more Ac-PGP when incubated with intact collagen *ex vivo*; this effect is inhibited by an ERK1/2 pathway inhibitor. Finally, clinical samples from individuals with CF demonstrate a notable correlation between Ac-PGP (as measured by liquid chromatography-tandem mass spectrometry) and MMP-9 levels even when accounting for total PMN burden.

**Conclusions/Significance:**

These data indicate that ECM-derived Ac-PGP could result in a feed-forward cycle by releasing MMP-9 from activated PMNs through the ligation of CXCR1 and CXCR2 and subsequent activation of the ERK1/2 MAPK, highlighting for the first time a matrix-derived chemokine (matrikine) augmenting its generation through a discrete receptor/intracellular signaling pathway. These findings have notable implications to the development unrelenting chronic PMN inflammation in human disease.

## Introduction

Matrix metalloproteases (MMPs) are members of zinc-dependent proteases responsible for degradation of extracellular matrix (ECM) components including basement membrane collagen, interstitial collagen, fibronectin, and various proteoglycans during normal remodeling and repair processes [1]. This family of proteolytic enzymes play a role in disease processes including various hematologic disorders, ocular disease, arthritis, cardiovascular diseases, infectious diseases and cancer [2]. MMP-9 (Gelatinase B, 92-kD type IV collagenase, EC 3.4.24.35) is an MMP that is present in low quantities in the healthy adult lung, but much more abundant in several chronic neutrophil (PMN)-predominant inflammatory lung diseases such as chronic obstructive pulmonary disease (COPD) and cystic fibrosis (CF). Although many intrinsic lung cells can be stimulated to produce MMP-9, inflammatory cells are thought to be the primary source of MMP-9 in disease [3]. PMNs are thought to play an important role in the secretion of MMP-9, which is released following stimulation by inflammatory mediators [4]. Excessive or inappropriate secretion of MMP-9 may contribute to the pathogenesis of tissue destructive processes in a wide array of diseases. High levels of MMP-9 related to the pathogenesis of lung injury have been demonstrated in CF [5] and COPD patients [6], [7], [8]. MMP-9 plays an important role in tissue destruction and inflammation through degradation of matrix proteins and proteolytic activation of cytokines/chemokines [9].

One family of ligand which plays an important role in neutrophilic disorders is the cysteine-x-cysteine (CXC) chemokines. The subfamily of CXC chemokines which are active on PMNs possess a Glu-Leu-Arg motif and are thus identified as ELR-positive CXC chemokines; these ligands act on specific CXC receptors on PMNs (CXCR1 and CXCR2) to induce PMN chemotaxis [10]. In humans, these include interleukin-8 (IL-8) and NAP-2 (active on CXCR1 and CXCR2 chemokine receptors) and the growth-related oncogene (GRO)-α, β and γ chemokines, which ligate only CXCR2 [11]. IL-8 is a potent PMN chemoattractant and a granulocytosis-promoting protein [12]. Besides the chemoattractant activity on PMNs, IL-8 triggers PMNs to release the contents of some of their granules [13]. Chakrabarty et al. demonstrated that IL-8 binding to CXCR2 causes the release of MMP-9 from tertiary granules [14] suggesting that PMN chemoattractants can augment protease production in disease.

In 1995, Pfister and colleagues demonstrated that alkali degradation of whole cornea generated two tri-peptides, N-acetyl-Pro-Gly-Pro (Ac-PGP) and N-methyl-PGP, which are chemotactic for PMNs and result from hydrolysis of collagen [15]. Ac-PGP shares both sequence and structural homology with an important domain of ELR-positive CXC chemokines, and competitively binds CXC chemokine receptors and requires CXCR1 and CXCR2 for its PMN chemoattractant activity [16]. Ac-PGP has been recognized to be a key chemoattractant in inflammatory diseases and seems to play as important of a role in PMN influx in chronic inflammatory conditions as traditional ELR+ CXC chemokines [17] and has recently been reported in the serum of CF patients [18]. MMP-9 is involved in the generation of Ac-PGP [19], [20], although it is unknown if Ac-PGP may induce MMP-9 release from PMNs, thereby potentially augmenting chronic PMN inflammation.

The goal of this study is to determine if Ac-PGP-induced granule release could result in a feed-forward cycle of inflammation by causing release of MMP-9, which is required for the ongoing production of Ac-PGP, and to subsequently reveal the intracellular signaling pathway involved in PMN activation by Ac-PGP. We find that Ac-PGP induces MMP-9 release from human PMNs via CXCR1 and CXCR2 receptor ligation. The activation of extracellular signal-regulated kinase (ERK)1/2 MAPK is required for release of MMP-9 by these cells. The impact of the ERK activation for MMP-9 mediated release is determined by mitigating MMP-9 release and inhibition of ex-vivo Ac-PGP generation via an ERK1/2 pathway inhibitor. Finally, clinical samples from individuals with CF, a chronic neutrophilic disorder, demonstrate a strong correlation in Ac-PGP stimulation and MMP-9, suggesting that this process may serve as an important switch to a self-propagating inflammatory state in a myriad of disorders.

## Results

### Time and dose-dependent increase in MMP-9 activity from Ac-PGP stimulated human PMNs

**Figure 1:**
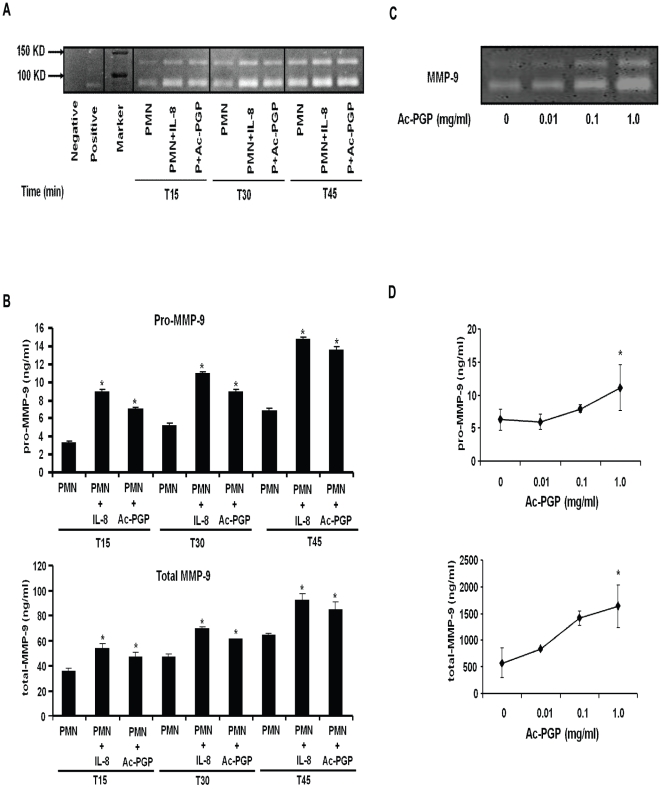
Increased MMP-9 activity in culture supernatants from human PMNs stimulated with Ac-PGP. Human PMNs isolated from peripheral blood were stimulated with Ac-PGP and IL-8 for different times and then supernatants were collected for MMP-9 assay. **A**. The detection of gelatinolytic activity in a time-dependent manner by g*elatin zymography* representative of three gels. **B**. The quantification of specific MMP-9 activity in a time-dependent manner by ELISA-based assay. **C**. The detection of gelatinolytic activity in a dose-dependent manner by g*elatin zymography* representative of three gels. **D**. The quantification of MMP-9 activity in a dose-dependent manner by ELISA-based assay. *p<0.05, compared with PMN only within same time point.

Since PMNs are a major cell type responsible for production of MMP-9, which is required for the generation of Ac-PGP, the potential role of Ac-PGP in a feed-forward cycle of MMP-9 production was investigated by examining whether Ac-PGP induces MMP-9 release in PMNs in vitro. Freshly isolated human PMNs were stimulated for 0, 15, 30, and 45 minutes with 1.0 mg/ml Ac-PGP and 1.0 μg/ml IL-8. These concentrations were selected as they reflect the relative potency for neutrophil chemotaxis for each of these molecules[16]. The cell-free supernatants were then collected and assayed for the presence of MMP-9 by gelatin zymography and a specific assay for MMP-9. We found that Ac-PGP, similar to IL-8, increases gelatinolytic activity in supernatants of PMNs in a time-dependent manner (Figure 1A). As shown in Figure 1B, the MMP-9 activity is elevated in Ac-PGP stimulated PMNs compared to the cells without stimulation. In addition, a dose-dependent increase in MMP-9 release was observed after Ac-PGP stimulation in both gelatinolytic activity (Figure 1C) and MMP-9 activity assay (Figure 1D). These results suggest that Ac-PGP, generated from the breakdown of collagen by MMP-9 protease, feeds back to induce MMP-9 release from PMNs to enhance ongoing inflammation.

### A specific ERK pathway inhibitor (U0126) blocks MMP-9 release in PMNs stimulated with Ac-PGP

**Figure 2:**
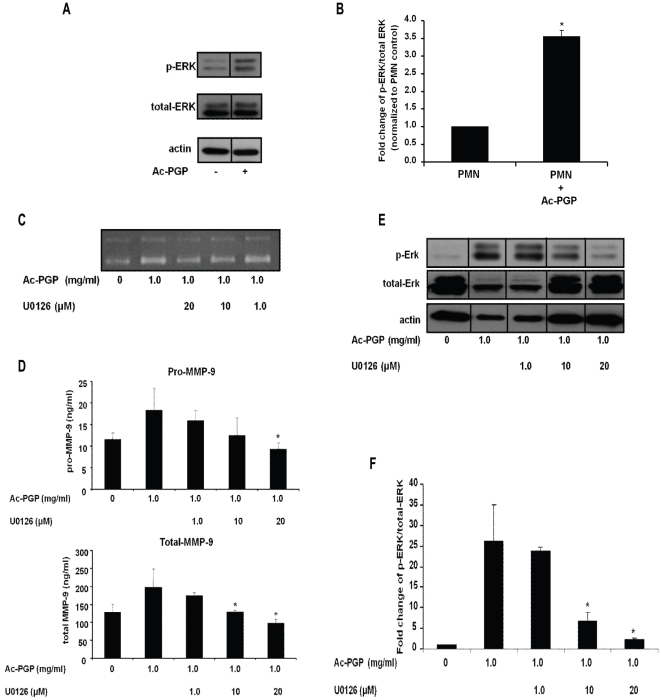
The effect of ERK1/2 MAPK inhibitor on the Ac-PGP mediated MMP-9 release in PMNs. PMNs were pretreated with ERK1/2 MAPK inhibitor U0126 or vehicle for 30 minutes, and then stimulated with Ac-PGP (1.0 mg/ml) for 30 minutes. Cell-free supernatants were collected for MMP-9 assay. The levels of ERK1/2 MAPK were determined by western blot analysis of lysates from stimulated PMNs with actin controls which paralleled total ERK 1/2. Phosphorylated ERK was determined using an anti-ERK antibody that recognizes phosphorylated threonine and tyrosine residues (Thr202/Tyr204). **A**. Total and phosphorylated level of ERK1/2 MAPK representative of three gels. **B**. Fold change of phosphorylation of ERK1/2 MAPK versus total ERK1/2 MAPK normalized to PMN control. **C**. The detection of gelatinolytic activity by g*elatin zymography* representative of three gels. **D**. The quantification of specific MMP-9 activity by ELISA-based assay. **E**. Total and phosphorylated level of ERK1/2 MAPK representative of three gels. **F**. Fold change of phosphorylation of ERK1/2 MAPK versus total ERK1/2 MAPK normalized to PMN control. *p<0.05 compared to Ac-PGP without inhibitor pretreatment.

MAPKs are regarded important mediators of a variety of physiological and pathological cellular processes, including cell death, cell survival, proliferation, and migration [21]. It has been reported that IL-8-induced MMP-9 release from PMNs involved the ERK1/2 kinase signaling pathway [14]. Since Ac-PGP demonstrated homology and utilizes the same receptors to induce chemotaxis of PMNs as IL-8, the potential of the MMP-9 release utilizing an ERK-mediated pathway was examined. To determine whether phosphorylation of ERK1/2 MAPK was involved in MMP-9 induction, PMNs were treated with Ac-PGP for 30 min, and then these cell lysates were analyzed using antibodies specific for the phosphorylated active forms of ERK1/2 MAPK by Western blot. As shown in Figure 2A and 2B, there was no significant difference in the level of total ERK1/2 and β-actin between protein extracts from PMNs. The levels of phosphorylated ERK1/2 were, however, four-fold higher in Ac-PGP stimulated PMNs compared with unstimulated PMNs. As a control, Jun N-terminal kinase (JNK) was examined and showed no difference in ratio of phosphorylated and total JNK with Ac-PGP stimulation (data not shown). These results suggested that Ac-PGP induces the activation of ERK1/2 MAPK in PMNs.

Based on the results of ERK1/2 activation (Figure 2A and 2B), the importance of the ERK1/2 pathway of MMP-9 release was interrogated utilizing a specific inhibitor for this intracellular cascade. It was well known that activation of ERK was mediated through an upstream component of a specific MAPK kinase, mitogen-activated protein kinase/extracellular signal-regulated kinase (MEK)1/2. Therefore, activation of ERK was further investigated using a highly specific MEK1/2 inhibitor, U0126. Pretreatment with U0126 significantly attenuated Ac-PGP-mediated MMP-9 release, which was demonstrated in both zymographic analysis (Figure 2C) and MMP-9 activity assay (approximately 66% reduction; P<0.05, as compared with that of Ac-PGP stimulated PMNs)(Figure 2D), suggesting that a link between activation of the ERK1/2 MAPK pathway and MMP-9 release induced by Ac-PGP. As expected, this Ac-PGP-mediated stimulation was accompanied by ERK1/2 phosphorylation and dose-dependent blockade with U0126 (Figure 2E and 2F). These results demonstrated the crucial role of ERK1/2 in distinct aspects of the Ac-PGP mediated MMP-9 release process in PMNs.

### Blockade of CXCR1/2 receptor inhibited MMP-9 production in PMNs stimulated with Ac-PGP

**Figure 3:**
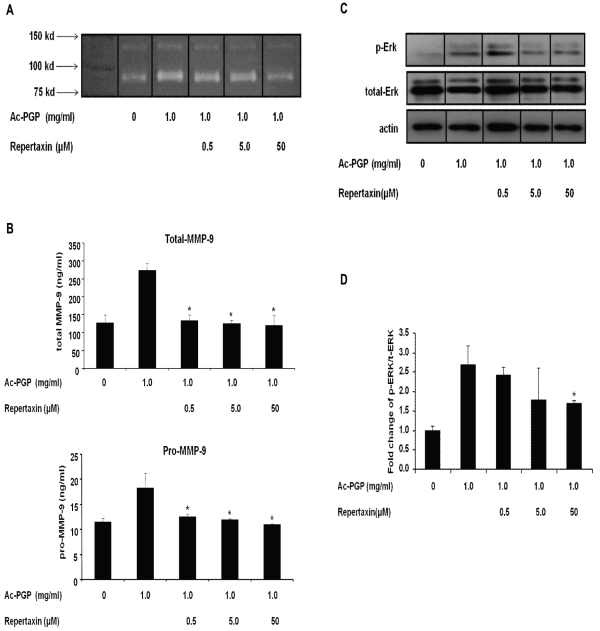
The effect of CXCR1 and CXCR2 inhibitor on the Ac-PGP mediated MMP-9 release in PMNs. PMNs were pretreated with the CXCR1 and CXCR2 inhibitor repertaxin at different dose for 20 minutes and then stimulated with Ac-PGP (1.0 mg/ml) for 30 minutes. Cell-free supernatants were collected for MMP-9 assay. The levels of ERK1/2 MAPK activation were determined by western blot analysis of lysates from stimulated PMNs with actin controls which paralleled total ERK 1/2. Phosphorylated ERK1/2 MAPK was determined using the anti-ERK antibody that recognizes phosphorylated threonine and tyrosine residues (Thr202/Tyr204). **A**. The detection of gelatinolytic activity by g*elatin zymography* representative of three gels. **B**. The quantification of MMP-9 activity by ELISA-based assay. **C**. Total and phosphorylated level of ERK1/2 MAPK representative of three gels **D**. Fold change of phosphorylation of ERK1/2 MAPK versus total ERK1/2 MAPK normalized to PMN control. *p<0.05 compared to Ac-PGP without inhibitor pretreatment.

Ac-PGP competitively binds CXC chemokine receptors and CXCR1 and CXCR2 ligation is required for its PMN chemotactic activity [16]. To determine the importance of the CXCR1 and CXCR2 receptor on the Ac-PGP mediated MMP-9 induction from PMNs, repertaxin (an inhibitor of CXCR1 and CXCR2) was used. Pretreatment of PMNs with repertaxin suppressed MMP-9 release from PMNs as shown in zymographic analysis (Figure 3A) and MMP-9 activity assay (Figure 3B). In order to demonstrate the importance of CXCR1 and CXCR2 signaling on ERK activation, PMNs (either stimulated with Ac-PGP or blocked with repertaxin) were examined for phospho- and total ERK expression. Figure 3C and 3D demonstrate that repertaxin blockade mitigates ERK phosphorylation, highlighting an important link between cell surface receptor activation and a discrete intracellular signaling pathway leading to MMP-9 release. These results suggested that Ac-PGP-induced MMP-9 release occurs via a CXCR-ERK activation signal pathways.

### Heavy labeled Ac-PGP produced collagen-derived Ac-PGP via an ERK-dependent pathway

**Figure 4:**
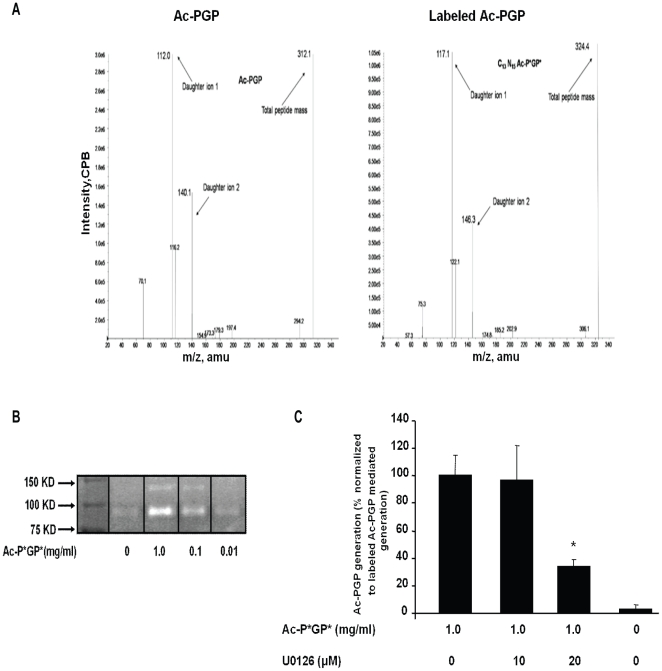
The blockade of ERK1/2 MAPK activation inhibited Ac-PGP production. Human peripheral blood PMNs (5.25×10^5^) were pretreated with U0126 (10 and 20 µM) versus vehicle for 30 minutes and then incubated for 30 minutes with labeled Ac-PGP. The supernatants collected from incubated cells were placed on type I collagen (1.0 mg/ml) for 24 hours at 37°C. Ac-PGP and C^13^N^15^ labeled Ac-PGP were analyzing by *ESI-LC-MS/MS*. Ac-PGP values of the samples on PBS were subtracted from Ac-PGP values of samples incubated on type I collagen to determine Ac-PGP production. **A**. Detection of Ac-PGP and C^13^N^15^ Ac-PGP via mass spectrometry. **B**. The detection of gelatinolytic activity in culture supernatants from human PMNs stimulated with labeled Ac-PGP by g*elatin zymography* representative of three gels. **C**. The measurement of Ac-PGP production by *ESI-LC-MS/MS.* The bar graph represents the percent of relative Ac-PGP production normalized to labeled Ac-PGP control. *p<0.05 compared to labeled Ac-PGP without inhibitor pretreatment, n = 4 wells/condition.

Since Ac-PGP has the ability to release MMP-9 from primary blood PMNs and MMP-9 is central to Ac-PGP generation, supernatants from Ac-PGP stimulated PMNs were studied to determine if they could generate de-novo Ac-PGP from intact collagen. In order to answer this question, a C^13^ N^15^ labeled Ac-PGP (with the heavy label placed on both prolines) was synthesized to distinguish it from Ac-PGP generated endogenously from collagen (Figure 4A). Spectra from Ac-PGP demonstrates a total peptide mass of 312, with daughter ions of 112 and 140 (Figure 4A, left panel); Spectra for C^13^N^15^ Ac-PGP show a peptide mass of 324 with daughter ions of 117 and 146 (Figure 4A, right panel). To determine if this heavy-labeled Ac-PGP (denoted as Ac-P*GP*) could release MMP-9 from PMNs, primary blood PMNs were stimulated with Ac-P*GP* and showed a similar increase in gelatinolytic bands with dose-dependent stimulation as unlabeled Ac-PGP (Figure 4B). Finally, supernatants from Ac-P*GP* stimulated PMNs generated increased endogenous Ac-PGP from collagen and this effect was mitigated by the pre-treatment of the U0126 inhibitor (Figure 4C). These findings further demonstrated the importance of ERK-mediated pathways in Ac-PGP generation and suggested important regulatory roles in human disease.

### Increased airway Ac-PGP correlated to high levels of MMP-9 and elevated numbers of PMNs

**Figure 5:**
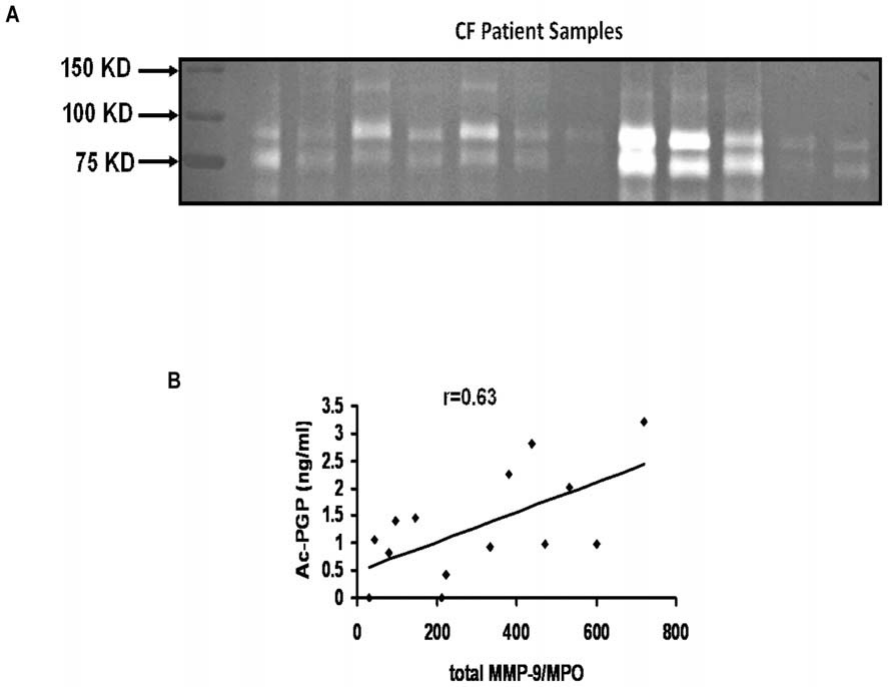
Ac-PGP correlated to the MMP-9/MPO ratio in CF patients. CF (*n = *14) sputum samples were collected to determine gelatinolytic activity by g*elatin zymography* (each lane represents an individual patient) (**A**) and for the measurement of Ac-PGP, MPO and MMP-9, and correlation analysis was conducted between Ac-PGP and MMP-9/MPO ratio (**B**). The CF sputum samples demonstrated a correlation coefficient (*r*) of 0.63 (p = 0.017).

It has been reported that there are increased levels of Ac-PGP in CF [19] and COPD [8] sputum suggesting an important role of this peptide in conditions with prominent ECM remodeling and neutrophilic inflammation. To determine whether Ac-PGP may be playing a role in MMP-9 release from PMNs *in vivo*, we attempted to analyze the correlations between Ac-PGP and high levels of MMP-9 in the CF lower airway. Sputum from 14 patients with inpatient CF exacerbation (average forced expiratory volume 1 second (FEV1)  = 39% (+/−24.1); 56%: delta F508 homozygotes, 22%: delta F508 heterozygotes, 22%:other genotype) were collected and analyzed for both MMP-9 and Ac-PGP peptides. Clinical data for this population is presented in Table 1. Zymography was done on samples (Figure 5A) showing notable but variable gelatinolytic activity in the samples. The samples were then assessed for a potential regulatory relationship between Ac-PGP and MMP-9 release in human disease. In order to control for the total PMN burden in a given sample as a confounder for increased MMP-9 expression, myeloperoxide (MPO), a marker of PMN influx, was measured. Figure 5B demonstrates that even when MMP-9 is controlled for by MPO, there is a notable correlation between Ac-PGP levels and MMP-9. These results demonstrate a strong correlation (r = 0.63, p = 0.017) in CF clinical samples between concentration of Ac-PGP and the level of MMP-9, recapitulating with fidelity our *ex vivo* and *in vitro* data.

**Table 1:** Clinical Data of CF Subjects.

Sample	Gender	Ageyears	Genotype	Bacteriology	FEV1%	FVC%	FEF(25–75%)
1	M	26	DeltaF508/17171G->A	PA^+^	22	34	9
2	F	34	DeltaF508/DeltaF508	PA^+^	39	52	19
3	M	23	Unknown/E60X	PA^+^	21	44	9
4	M	32	Unknown/Unknown	SA^+^,PA^+^	19	26	7
5	M	20	DeltaF508/DeltaF508	PA^+^	34	60	8
6	F	11	DeltaF508/DeltaF508	PA^+^	104	100	102
7	F	23	DeltaF508/DeltaF508	SA^+^	60	56	57
8	M	30	Unknown/DeltaF508	PA^+^	37	43	21
9	M	20	DeltaF508/DeltaF508	PA^+^	25	36	10
10	F	33	DeltaF508/3849+10KBC->T	SA^+^, PA^+^,few AX	24	31	14
11	M	34	DeltaF508/DeltaF508	SA^+^,PA^+^	34	49	31
12	M	31	DeltaF508/DeltaF508	SA^+^,PA^+^	28	42	9
13	F	23	DeltaF508/DeltaF508	SA^+^	71	69	60
14	M	20	Unknown/Unknown	SA^+^,PA^+^	24	43	6
N = 14	36% females64% males	25±6.9 (M±SD)	56% Delta F508 homozygous22% Delta F508 heterozygous22% other or unidentified	50% PA^+^ only14% SA^+^ only36% PA^+^ & SA^+^	38±24.1(M±SD)	49±18.8(M±SD)	26±28.2(M±SD)

## Discussion

An important question in the field of inflammatory biology is how a self-limited, acute neutrophilic inflammatory pathway in response to a specific stimulus can become a self-propagating, chronic inflammatory response when the initial inciting event is often resolved [22]. While chemokine pathways are thought to play an important role in the initial inflammatory response seen to stimuli, their role in a persistent neutrophilic inflammatory state is not well-defined. This apparent disparity in the roles of preformed chemokine mediators in acute and chronic neutrophilia has led many to hypothesize that there are other discrete pathways involved in chronic neutrophilic inflammatory response. Our initial description of Ac-PGP highlighted this novel fragment's capability to cause PMN chemotaxis by ligation of CXC receptors [16]. Despite this exciting finding of a non-cognate ligand for CXC receptors residing in the extracellular matrix (ECM), it did not explain how this matrikine could serve to induce a shift from an acute to chronic neutrophilic inflammatory phenotype.

In a previous study, we demonstrate that the release of MMP-9 into the airway leads to the cleavage of collagen fragments, leading to Ac-PGP generation. MMP-9 is a protease that degrades extracellular matrix proteins including gelatin, collagen, elastin, and laminin [14], [23] and plays important roles in tissue destruction and also in tissue remodeling and inflammation [24]. Recent evidence suggests that although PGP is present in human disease specimens, autoantibodies directed at the fragments are not observed [25]. Here, for the first time, we describe how Ac-PGP can stimulate primary peripheral blood PMNs and induce the release of MMP-9 from PMN tertiary granules. This effect is observed in a time dependent manner, examining MMP-9 activity (Figure 1A) and quantity of MMP-9 (Figure 1B) from 15 to 45 minutes of Ac-PGP stimulation. In addition, a dose-dependent increase in both MMP-9 activity (Figure 1C) and quantity (Figure 1D) was observed over a three-log increase in Ac-PGP concentration. These findings highlight that, similar to IL-8, Ac-PGP can act to induce MMP-9 release in a specific, coordinated manner from PMNs.

MMP-9 has been observed to be differentially regulated during situations of acute versus chronic neutrophilic responses [26]. Previous evidence has shown that various intracellular pathways may be important in the regulation of MMP-9 expression during inflammation. Several lines of evidence have shown that MMP-9 gene expression is regulated by the p42/p44 MAPK, p38 MAPK, and JNK in different cell types [27], [28], [29], [30], [31]. Indeed, the MAPK pathways mediate diverse functions such as cell division and degranulation in many cell types [32], [33], [34]. Human PMNs express ERK1/2, JNK, and p38 MAPKs [35], [36], and ERK1/2 is activated following stimulation with IL-8 [37]. Activation of ERK1/2 regulates various effects in PMNs and other granulocytes including release of granule contents by degranulation [38], [39]. In the present studies, we demonstrate that activation of the ERK1/2 MAPK pathway is necessary for MMP-9 release by PMNs in response to Ac-PGP. This is supported by the finding that Ac-PGP specifically phosphorylated and activated the ERK1/2 MAPK pathway (Figure 2A and 2B). This ERK1/2 activation seen intracellularly from Ac-PGP stimulated PMNs corresponds with increases in MMP-9 activity (Figure 2C) and quantity (Figure 2D) from supernatants, suggesting a potential link between Ac-PGP,ERK1/2, and MMP-9 release. To confirm the central importance of the ERK1/2 MAPK pathway in MMP-9 release, a specific cell-permeant ERK1/2 inhibitor, U0126, is utilized and successfully blocked intracellular ERK phosphorylation (Figure 2E and 2F) and Ac-PGP-induced MMP-9 release in PMNs, in a dose-dependent manner (Figure 2C and 2D) confirming that Ac-PGP induces the release of MMP-9 from PMNs through an ERK MAPK pathway.

Since the blockade of CXCR1 and CXCR2 receptors can suppress the degranulation of PMNs induced by different chemokines and cytokines [14], [40], [41] and Ac-PGP acts through CXCR1 and CXCR2-dependent mechanisms to attract PMNs to inflammatory sites [16], we also examined the impact of CXC blockade on the MMP-9 release axis. The ERK1/2-mediated release of MMP-9 from PMNs (Figure 3A and 3B) and intracellular ERK1/2 phosphorylation (Figure 3C and 3D) are significantly abrogated when repertaxin, a CXCR1 and CXCR2 specific inhibitor, was utilized. Collectively, these findings indicate a crucial inflammatory connection of external receptor activation and internal signaling through an Ac-PGP-CXCR-ERK1/2-MMP-9 pathway, strongly suggesting a novel feed-forward pathway to augment Ac-PGP production.

A recent article has suggested the possibility that Ac-PGP does not directly interact with CXC receptors on PMNs to cause chemotaxis [42]. This was in large measure due to this group's inability to demonstrate competition of Ac-PGP with radiolabeled IL-8 in a CXCR radioreceptor assay. In all likelihood, this effect was observed due to not preincubating a low affinity CXCR ligand (Ac-PGP) with PMNs prior to incubation with the higher affinity ligand (IL-8) in the radioreceptor assay. In addition, these authors claim that Ac-PGP does not activate G-proteins but it is difficult for us to see how this would be possible since Ac-PGP is clearly chemotactic for PMNs as demonstrated in this group's manuscript as well as others [16], [19], [42], [43] and PMN chemotaxis is mediated through G-protein coupled receptors and activation of G-proteins.

Figure 4 demonstrates that, by utilizing a heavy-labeled Ac-PGP as a PMN stimulus, Ac-PGP can be generated in an *ex vivo* assay and this effect is blocked by the ERK1/2-specific inhibitor thereby definitively showing that Ac-PGP has the capability to induce its own generation through a PMN-mediated pathway. This pathway may play an important role in augmentation of ongoing neutrophilic inflammation observed in chronic inflammatory conditions. Our group has previously demonstrated that individuals without known lung disease have very low MMP-9 activity, Ac-PGP levels, and PMN burden [5], [19] suggesting that there may be dysregulation of important protease-mediated inflammatory mechanisms in chronic neutrophil-predominant lung disorders such as CF. To examine this possibility, sputum samples from CF patients were examined for Ac-PGP content and MMP-9 content in the hopes of finding a potential correlation in a chronic PMN –predominant human disease. Since MMP-9 levels may be increased solely by the presence of increased PMN burden in the clinical sample, the MMP-9 level is controlled for by the MPO levels (a validated surrogate marker for PMN influx) in the clinical samples. Despite controlling for PMN burden, a very strong correlation (r = 0.63, p = 0.017) is observed between MMP-9 and Ac-PGP strongly suggesting that the regulatory relationship observed in the experimental models may also be important in a chronic inflammatory disorder.

**Figure 6:**
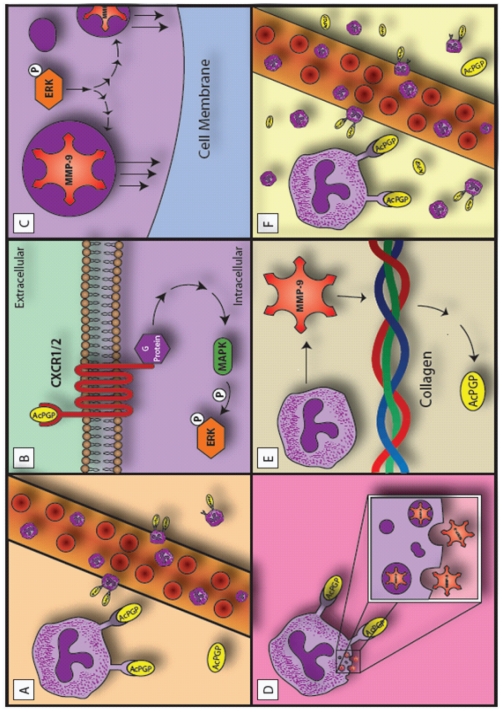
A model of persistent matrikine production and neutrophilic inflammation. During chronic PMN inflammation, collagen is hydrolyzed releasing Ac-PGP causing ongoing neutrophilic influx (**A**). In addition to causing PMN influx, Ac-PGP ligation of CXCR1 and CXCR2 leads to intracellular ERK phosphorylation and activation (**B**) and degranulation of MMP-9 from PMN tertiary granules (**C and D**). This MMP-9 acts on exposed collagen leading to Ac-PGP generation (**E**) and a feed-forward inflammatory response on PMNs (**F**).

To our knowledge, this work represents the first data demonstrating that a matrikine (such as Ac-PGP) has the capacity to induce its own generation, thereby augmenting an inflammatory response. Based on the observations from our previous studies and current findings, a notable “feed-forward” model of Ac-PGP inflammation is very apparent. It is possible that recruited PMNs from blood may bind either chemokines or Ac-PGP via CXCR1 and CXCR2, leading to intracellular ERK activation and subsequent MMP-9 release. MMP-9 then leads to ongoing collagen hydrolysis with Ac-PGP production. This pathway forms a positive feed-forward loop between Ac-PGP, PMNs, and MMP-9 (Figure 6). As such, there are numerous potential therapeutic targets in this system of chronic neutrophilic inflammation; perhaps the most appealing is blocking generation of Ac-PGP by inhibition of MMP-9 either in the extracellular environment or by prevention of release through targeting CXC receptors. Recently, our group has also shown that PGP has an endogenous anti-inflammatory pathway mediated by leukotriene A4 hydrolase and regulating this enzyme may also offer a unique treatment pathway [44]. Currently, our laboratory is actively pursuing compounds which focus on these and other regulatory pathways in the generation of chronic neutrophilic inflammation.

## Materials and Methods

### Patient Populations and Sputum Collection

All human studies were approved by the UAB Institutional Review Board. CF patients were recruited from the UAB Gregory Fleming James Cystic Fibrosis Center. All subjects carried the diagnosis of CF based on accepted diagnostic criteria [45]. All subjects provided written informed consent. Samples and health information were labeled using unique identifiers to protect subject confidentiality. Sputum samples were spontaneously expectorated by subjects. Sputum samples were collected on ice, diluted 1∶1 with 1X PBS, centrifuged at 1000 rpm for 15 min, and supernatants were stored at -80°C for later analysis.

### Reagents

Ac-PGP was synthesized by Anaspec Inc., (San Jose, USA). RIPA lysis buffer, PMSF, Sodium Orthovanadate, Albumin from bovine serum (cohn fraction V) are from Santa Cruz (Santa Cruz, CA USA). Ficoll-Paque was from GE Healthcare Bio-Sciences Inc., (Montréal, Canada). HBSS without phenol was from Mediatech, Inc. (Hernden, VA). Rabbit anti-human ERK1/2 antibody, rabbit anti-phospho-human ERK1/2 antibody (DB.14.4E), rabbit anti-human total and phospo-JNK antibodies, goat anti-rabbit coupled to HRP, and U0126 were purchased from Cell Signaling Technologies (Beverly, MA, USA). Chemiluminescence staining kit was from Millipore Corp. (Billerica, MA, USA). MMP-9 -specific ELISA kits and recombinant human (rh) IL-8 were purchased from R&D Systems Inc. (Minneapolis, MN, USA). MPO kits were purchased from Calbiochem (San Diego, CA, USA). β-actin antibody (Clone Ac-15), type I collagen, and gelatin was purchased from Sigma-Aldrich (St. Louis, MO, USA).

### Treatment of PMNs with Ac-PGP

For blood PMNs preparation, venous blood was separated on Ficoll (1.119 g/ml, bottom) and Ficoll-Paque (1.077 g/ml, top) layers to separate the granulocytes from the mononuclear leukocytes. The granulocyte pellet was washed in HBSS two times and then resuspended in HBSS containing 1% BSA (w/v). The purity of PMN preparations were 95% via staining and flow cytometry.

The prewarmed PMN suspensions (37°C, 3.5×10^6^ cells/ml) were incubated in 1% BSA/HBSS with or without Ac-PGP (0.01–1.0 mg/ml) or IL-8 (1.0 µg/ml) for different time intervals at 37°C in 5% CO2. For all treatment conditions described in these experiments and all time points studied, PMN viability was assessed by trypan blue exclusion in all conditions, doses, and time points, and the PMN viability was >97%, which correlated with cell viability observed for time periods less than 2 hours ex vivo [46]. The level of gelatinolytic enzymes released in cell-free supernatant was measured by gelatin zymography and the MMP-9 activity in cell-free supernatant was measured by an ELISA-based assay. For the analysis of MMP-9 release inhibition, the prewarmed PMN suspensions were treated for 20 to 30 minutes with the inhibitors (or vehicle) at the indicated concentrations (see figure legends), followed by an incubation with or without Ac-PGP (1.0 mg/ml) for 30 minutes at 37°C in 5% CO_2_. Cell-free supernatant were then harvested for gelatinolytic enzymes release and MMP-9 assay.

### Zymography

Porcine skin gelatin at 1.0 mg/ml was added to a 7.5% SDS-polyacrylamide solution before casting. The samples were diluted in nonreducing sample buffer, and 25 µl of sample was added to each lane. All samples are electrophoresed at 45 V for 5 h at 4°C. Following electrophoresis, gels were washed in 2.5% Triton X-100 for 30 minutes at 25°C, and then incubated in 50 mM Tris and 5.0 mM CaCl (pH = 8.0) for 16 h at 37°C. Gels were stained in 0.05% Coomassie blue for 30 min and subsequently distained in acetic acid and methanol for optimal exposure. Higher molecular weight bands on gelatin zymograms likely represented MMP-9/NGAL complexes.

### MMP-9 activity assay

MMP-9-specific ELISA-based activity assays were used to quantify specific MMP activity. Both samples and recombinant enzyme standards were prepared and incubated for 2 h at room temperature in 96-well plates coated with a mAb for MMP-9. After incubation, samples and standards were activated with 1.0 mM 4-aminophenylmercuric acetate (APMA), a chemical activator of MMPs, and further incubated for 2 h at 37°C. After incubation, a fluorogenic substrate (Fluor-Pro-Leu-Gly-Leu-Ala-Arg-NH2) was placed in each well and the plate was incubated at 37°C for 18 h. The plate was then read on a spectrophotometer (excitation and emission wavelength of 320 and 405, respectively, SpectraMax Gemini; Molecular Devices) and data were quantified using standard curves provided with the kits. The wells not treated with APMA represent the pro-MMP-9 level in the sample whereas the matched wells treated with APMA represent the total MMP-9 level (pro+activated) in the sample.

### Myeloperoxidase (MPO) assay

The Calbiochem® InnoZymeTM Myeloperoxidase Activity Kit was used to quantify specific active human MPO. Both samples and MPO standards were prepared and incubated for 1 h at room temperature with gentle shaking in 96-well plates coated with polyclonal antibody specific for human MPO. After four washes, detection reagent that included TMB and hydrogen peroxide was added for 30 minute incubation at 37°C. Following color development, the reaction was stopped with sulfuric acid and the absorbance of the oxidized TMB is detected at 450 nm. The concentration of samples was determined by interpolation from the standard curve.

### Analysis of ERK-1/2 MAPK activation by Western blot

PMNs were incubated with Ac-PGP (1.0 mg/ml) for 30 minutes. To evaluate the efficacy of the ERK pathway inhibitor, U0126, to inhibit ERK1/2 phosphorylation and activation, PMNs were treated with the indicated concentrations of inhibitor in 1% BSA/HBSS for 30 min, followed by incubation with or without Ac-PGP (1.0 mg/ml) for 30 min. The samples were centrifuged, and the cell pellets were disrupted in cold (4°C) RIPA lysis buffer. After 30 min incubation on ice, the lysates were centrifuged to remove the debris, and the supernatants were then boiled for 5 min in Laemmli buffer. Samples (15 μg total protein/lane, unless otherwise stated) were then electrophoresed through 10% SDS-polyacrylamide gels and were transferred onto nitrocellular membranes, which were blocked in TBST (pH 7.4) containing 5% BSA for 1 h and probed with 1∶2000 anti-phospho-ERK-1/2 or anti-ERK-1/2, followed by the appropriate secondary antibody coupled to HRP (1∶5,000; Cell Signaling Technology, Beverly, MA, USA). Immunoblots were then developed using ECL chemiluminescent kits. Densitometric analyses were performed using NIH Imaging software.

### Electrospray ionization-liquid chromatrography-mass spec/mass spec (ESI-LC-MS/MS)

Ac-PGP and Ac-P*GP* were measured using a MDS Sciex (Applied Biosystems, Foster City, CA) API-4000 spectrometer equipped with a Shimadzu HPLC (Columbia, MD). HPLC was conducted using a 2.0×150 mm Jupiter 4u Proteo column (Phenomenex, Torrance, CA) with A: 0.1% HCOOH and B: MeCN +0.1% HCOOH: 0 min-0.5 min 5% buffer B/95% buffer A, then increased over 0.5–2.5 min to 100% buffer B/0% buffer A. Background was removed by flushing with 100% isopropanol/0.1% formic acid. Positive electrospray mass transitions occured at 312-140 and 312-112 for Ac-PGP and 324-146 and 324-117 for Ac-P*GP*.

### *Ex Vivo* Collagen Assay

Type I collagen (1.0 mg/ml) was solubilized by wetting with glacial acetic acid and diluting to volume with water (final pH<4.5), and rotated until complete dissolved. This solution was then extensively dialyzed against distilled water (pH adjusted to 3.5 by addition of acetic acid). 40 μl of cell-free supernatants were incubated with 40 μl of dialyzed intact type I collagen for 24 h at 37°C and 5% CO_2_ with exogenous prolyl endopeptidase (2.5 µg/ml). After incubation, samples were filtered through a 10-kDa filter (Millipore Corporation, Billerica, MA), the filters were washed with 40 μl of 1.0 mM HCl. The solutions were analyzed by multiple reaction monitoring (MRM) using ESI- LC-MS for levels of Ac-PGP. Amounts of Ac-PGP generated by each cell-free supernatant sample from collagen were determined by comparison with cell-free supernatant incubated with PBS.

### Statistical analyses

Descriptive statistics including mean and SD were conducted for all quantitative measures. The two-tailed Student *t* test was used for comparisons between two groups and ANOVA was used for comparing means of three or more groups. Spearman correlation was used to compare the relationship between Ac-PGP levels and MMP-9/MPO levels. The results were considered significant if *p* values were 0.05 or less.
